# Evaluating the impact of personalized rehabilitation nursing intervention on postoperative recovery of respiratory function among thoracic surgery patients: A protocol for systematic review and meta-analysis

**DOI:** 10.1097/MD.0000000000028776

**Published:** 2022-03-04

**Authors:** Ai Liu, Mian Li, Wenjin Gao, Xiaoke Wen, Hui Zhu, Yanyan Chen

**Affiliations:** a Department of Cardiothoracic Surgery, Wuhan Puren Hospital, Wuhan, Hubei Province, China; b Department of Neurosurgery, Wuhan Puren Hospital, Wuhan, Hubei Province, China; c Outpatient Department, Wuhan Puren Hospital, Wuhan, Hubei Province, China.

**Keywords:** personalized rehabilitation nursing, recovery, respiratory function, thoracic surgery

## Abstract

**Background:**

Owing to clinical developments and economic strain, perioperative care has undergone considerable changes. Therefore, it is important to review and critique the efficacy of existing practices in a context that is placing increasing emphasis on better efficacy and cost-containment. Considering that the objective involves devising approaches to minimize postoperative complications and reduce medical care, efforts should concentrate on postsurgical pulmonary complications that are common. The present analysis aims to examine how customized rehabilitation nursing intercession impacts the postsurgical restoration of respiratory functions in thoracic surgery patients.

**Methods:**

Prespecified search strategies will be employed to perform a methodological search of 6 databases namely EMBASE, Cochrane Library, PubMed, Web of Science, WanFang Database, and China National Knowledge Infrastructure. The analysis will comprise original publications that evaluated how personalized rehabilitation nursing intervention impacts postsurgical restoration of respiratory function in those who have undergone thoracic surgery. All considered publications are before December 25, 2021. Different authors will conduct an independent study selection process to evaluate the quality of the publications and extract required data. Based on the standardized mean difference and its 95% confidence interval, we estimate the summary effects for each meta-analyses. Based on heterogeneity in considered articles, the related data will be pooled through either a random- or fixed-effect meta-analysis. Lastly, the overall quality of evidence using appropriate methods will be performed.

**Results:**

The results of this analysis will systematically evaluate how customized rehabilitation nursing interference impact postsurgical healing of respiratory functions in patients who have undergone thoracic surgery by collecting the existing evidence.

**Ethics and dissemination:**

Not required.

**Open Science Framework registration number:**

10.17605/OSF.IO/NBVYW.

## 1 Introduction

Thoracic surgical process impedes postsurgical respiratory function, increasing the probability of the onset of postoperative pulmonary complications (PPCs). The incidence of PPC exceeds upper or lower abdominal operation.^[1,2]^ Thoracic surgical intervention can involve prolonged surgical durations, increased trauma, 1-lung ventilation, nerve injury, acute or chronic pain, and sternum or rib incision. Since the characteristics create numerous complications that adversely impact the prognosis of patients, surgeons and anaesthesiologists have increasingly studied PPCs of thoracic operations.^[3,4]^ In addition, prolonged hospitalizations and admissions to the intensive therapy unit are other significant medical and financial strains caused by PPCs. Risk factors associated with PPCs in the follow-up of pulmonary resection have been recognized in several clinical researches adopting various research designs and definitions. Accordingly, age, smoking status, presurgical pulmonary function assessments, chronic obstructive pulmonary disease, and cardiovascular comorbidity are the most frequent risk factors.^[5,6]^

High-quality nursing model is proposed and recognized by both doctors and nurses to improve the patients’ quality of life and their satisfaction with nursing work.^[7]^ Accordingly, patients undergoing critical thoracic surgery should avoid adverse effects on the heart, lungs, and other organs, and simultaneously prevent adverse reactions, such as blood circulation disorders and respiratory insufficiency, and require good care of the patients.^[8]^ It is necessary to identify factors likely to relate to appropriate nursing intervention to reduce adverse effects to improve the postsurgical restoration of respiratory function among thoracic surgery patients. Personalized rehabilitation nursing is the provision of clinical services with high standard to families to comprehend treatment effect, psychological state of the patients, and compliance behaviour of discharged patients and offer them clinical and psychological guidance to elevate their standard of life.^[9,10]^ Overall, the present study aims to evaluate how personalized rehabilitation nursing intervention impact postsurgical restoration of respiratory function in patients who have undergone thoracic surgery.

## 2 Methods

### 2.1 Design and registration of the review

The protocol complies with the preferred reporting items for systematic reviews and meta-analyses protocols guidelines.^[11]^ The current systematic review and meta-analysis protocol is registered under the Open Science Framework (http://osf.io/).

### 2.2 Inclusion criteria for study selection

#### 2.2.1 Types of studies

Observational studies presenting accessible data on how customized rehabilitation nursing intercession impact postsurgical restoration of respiratory function among patients who have undergone thoracic surgery shall be considered. Studies that will be excluded include animal research, case reports, and uncontrolled trials.

#### 2.2.2 Types of patients

All patients undergoing thoracic surgery will be eligible, irrespective of education status, race, and demographic age.

#### 2.2.3 Types of interventions

The analysis will include research articles that have administered personalized rehabilitation nursing to intervention groups, whereas any traditional nursing method was administered to control group.

#### 2.2.4 Outcome measures

Outcome measures encompass forced expiratory volume at 1 second, forced expiratory volume at 1 second/forced vital capacity, forced vital capacity, heart rate, respiratory frequency, nursing satisfaction, the incidence of adverse reactions, and serious events, and the hospitalization duration.

### 2.3 Search methods for the identification of studies

#### 2.3.1 Electronics searches

Prespecified search strategies will be employed to perform a methodological search of 6 databases, namely EMBASE, Cochrane Library, PubMed, Web of Science, WanFang Database, and China National Knowledge Infrastructure. The analysis will comprise original publications that evaluated how personalized rehabilitation nursing intervention impacts postsurgical restoration of respiratory function in those who have undergone thoracic surgery. All considered publications are before December 25, 2021. We will combine the following key words and search terms to search the databases: thoracic surgery, respiratory function, recovery, personalized rehabilitation.

#### 2.3.2 Search other resources

Additional databases will be utilized to salvage potential studies: Google Scholar, the NIH clinical registry ClinicalTrials.gov, and CINAHL. We would manually access the related meta-analyses and perform a review to classify additional publications.

### 2.4 Data collection and analysis

#### 2.4.1 Selection of studies

We will create a database in EndNote V.9 and upload all records obtained from databases and numerous resources. The review authors will autonomously screen extracted abstracts. We shall collect the complete text of all possibly appropriate studies to conduct an extended assessment of the eligibility according to the eligible/illegible criteria. Publications that do not satisfy the eligibility criteria shall be omitted and we will record the reason for excluding each study. All disagreements will be mediated through consensus or consulting another (third) author.

#### 2.4.2 Data extraction and management

Two authors will perform an independent application of the eligibility and illegibility criteria to evaluate the eligibility of each retrieved study. Subsequently, we will extract the following data from the chosen publications by employing a data collection form and Excel spreadsheet: study design, inclusion criteria, method of intervention, fundamental characteristics of included studies, outcome measures, reported adverse events, bias risk assessment, and findings. Two reviewers will cross-check the results, and disagreements shall be resolved through consensus or by consulting a third author.

#### 2.4.3 Assessment of risk of bias

A pair of autonomous authors will perform a bias risk assessment of the included studies, which will be as per the Cochrane Handbook guideline.^[12]^ We shall mediate all differences in opinion via consensus or consulting with another (third) author.

#### 2.4.4 Measures of treatment effect

We will use RevMan (v 5.3) to synthesize and statistically analyse the data. We shall employ a risk ratio with 95% confidence interval to analyse dichotomous data. Meanwhile, we will use the mean difference or standard mean difference with 95% confidence interval to analyse the continuous data. Standard mean difference if different evaluation tools are used.

#### 2.4.5 Assessment of heterogeneity

For each study, the effect sizes and corresponding 95% confidence limits will be presented using forest plots. Simultaneously, the *I* ^2^ test will be computed to quantify the inconsistency.

#### 2.4.6 Assessment of reporting biases

If there are over 10 studies, we will employ a funnel plot to observe the reporting biases generated.^[13]^ Egger test will be used to further assess any potential reporting biases.

#### 2.4.7 Sensitivity analysis

When applicable, a sensitivity analysis will be performed to investigate how the bias risk of the trial affect the primary outcomes. The sensitivity analyses shall omit trials of low quality before repeating the meta-analyses to evaluate the quality and robustness in the presence of significant statistical heterogeneity, primarily based on insufficient data and sample size.

## 3 Discussion

This systematic analysis summarizes high-quality evidence to investigate how personalized rehabilitation nursing intervention impact the postsurgical restoration of respiratory function in thoracic surgery patients. The study is a reference for nurses and helps develop clinical guidelines. As far as the authors are aware, this is the first systematic analysis to specifically concentrate on investigating how personalized rehabilitation nursing intervention impacts postsurgical restoration of respiratory function in patients who have undergone thoracic operations. An increasing number of studies have investigated how personalized rehabilitation nursing intervention impacts the postsurgical restoration of respiratory function in thoracic surgery patients. However, there is controversy in the findings. Thus, this study shall be carried out to perform a systematic investigation on how customized rehabilitation nursing interference impacts the postsurgical healing of respiratory functions in those who have received thoracic surgical intervention.

## Author contributions

**Conceptualization:** Ai Liu.

**Data curation:** Ai Liu, Mian Li, Wenjin Gao, Xiaoke Wen, Hui Zhu, Yanyan Chen.

**Formal analysis:** Ai Liu, Xiaoke Wen.

**Funding acquisition:** Mian Li, Wenjin Gao, Yanyan Chen.

**Investigation:** Ai Liu, Xiaoke Wen.

**Methodology:** Mian Li, Wenjin Gao, Xiaoke Wen, Hui Zhu, Yanyan Chen.

**Project administration:** Mian Li, Xiaoke Wen.

**Resources:** Ai Liu, Yanyan Chen.

**Software:** Ai Liu, Mian Li, Wenjin Gao, Xiaoke Wen, Hui Zhu.

**Supervision:** Mian Li, Hui Zhu, Yanyan Chen.

**Validation:** Ai Liu, Mian Li, Wenjin Gao.

**Visualization:** Mian Li, Hui Zhu, Yanyan Chen.

**Writing – original draft:** Ai Liu.

**Writing – review & editing:** Yanyan Chen.
